# Editorial: Psycho-physical stressors in youth sport performance

**DOI:** 10.3389/fpsyg.2024.1518655

**Published:** 2024-11-26

**Authors:** Valerio Bonavolontà, Cosme Franklim Buzzachera, Maria Chiara Gallotta

**Affiliations:** ^1^Department of Biotechnological and Applied Clinical Sciences, University of L'Aquila, L'Aquila, Italy; ^2^Department of Public Health, Experimental and Forensic Medicine, University of Pavia, Pavia, Italy; ^3^Department of Physiology and Pharmacology “Vittorio Erspamer”, Sapienza University of Rome, Rome, Italy

**Keywords:** youth sport, parental involvement, coaching, athletic development, stressor

The youth sport experience is a fundamental stage in the psychophysical development of children and adolescent as well as of their long-term athletic development across the lifespan.

Extensive research highlights, among the several factors influencing such a multi-dimensional construct, the crucial roles of parents and coaches in fostering positive and optimal youth sport practice and experience. The aim of this Research Topic was to identify possible psychophysical factors that can influence an effective youth development and can help developing strategies to improve the sport experience of young athletes.

The papers contained in this Research Topic aimed to contribute a small but insightful view of the youth sport performance scenario by considering some of the several well-recognized psychophysical stressors.

In their paper Gao et al. consider how parents can influence athletes' motivation, which is considered one of the key factors for young athletes, with a focused systematic review based on 29 studies and more than 9,000 and 2,000 participants among young athletes and parents, respectively.

Indeed, previous works have suggested that parents are primary social agents in sports that can influence young athletes' motivation more than coaches do (O'Rourke et al., 2014; Atkins et al., 2015; Amorose et al., 2016). The authors report that positive parental goals and values, parenting styles that support autonomy, moderate parental involvement, positive parent–child relationships, and parent-initiated task climate can be identified as optimal parenting strategies. These findings are in line with previous work by Bonavolontà et al. (2021), according to which excessive parental involvement can cause pressure on children, who would prefer parental involvement characterized by praise and understanding. Therefore, a balance between active and moderate parental involvement seems to be advisable for a successful parent-child relationship in relation to children's sports participation. Gao et al. conclude that while parents play an undeniable key role in the motivational process of young athletes, the manner of their involvement makes the difference.

The paper by McCabe et al. investigated young athletes' perceptions of their parents' sideline sport behavior and their own sporting behaviors by analyzing the linear regression between these variables. In this brief Australian research report, based on Social Learning Theory, the authors explore the relationship between parental and young athletes' behaviors, reporting that when youths perceived positive parental behaviors (e.g., cheering/clapping) they were associated with prosocial behaviors. Similarly, perceived negative parental behaviors (e.g., “how often has your parent encouraged you to hurt a player on the other team?”), were correlated with youth athletes' antisocial behaviors. Moreover, the paper reported a correlation between athletes' age and positive behaviors by their parents, suggesting that as youth become more independent, parents may change their support/involvement.

The meta-analysis by Lin et al., on the other hand, investigated the mediating role of self-efficacy in the relationship between social support (family support, peer support and school support) and physical activity (PA) in adolescents. The study, based on 56 studies with a large total sample, reported that social, peer and family support was positively correlated with adolescents' PA, regardless of their grade level, gender, economic status and cultural background while school support was not. Self-efficacy was also found to be correlated with adolescents' social support and PA, supporting the applicability of the ecologic theories of social support and self-efficacy, as it accounted for more than 30% of the total effect, suggesting a significative mediating role between the variables. Moreover, the effect of self-efficacy on adolescents' PA was reported to be stronger than social support and its subtypes, accounting for a relevant proportion of the influence of social support PA in adolescents. Interestingly, school support did not have a direct effect on adolescent PA, even if mediated by self-efficacy, which allows a reflection on how school environment and conditions may not be favorable to promote PA.

Thus, based on these results, the promotion of adolescents PA should consider the role of self-efficacy in the use of social support and should also emphasize the role of peer support in helping adolescents to motivate each other.

The last paper of this Research Topic analyzed the determinants of the dropout phenomenon among young scholars. Dropping out in youth sport is a relevant and well-investigated topic, which has been associated with early specialization, parental involvement, coaching styles and behaviors, but also with bullying, health issues, peer influence and lack of enjoyment. The cross-sectional study by Pisaniello et al., explored a large sample aged group 8–13 years old, the transition period from childhood to adolescence that is critical for sports abandonment, through a structured questionnaire that highlighted lack of time and high cost as the most relevant reasons contributing in dropping out, along with lack of motivation and interest, incompatible schedule or absence of facilities.

These findings confirm previous literature suggesting a broad multifactorial holistic approach to reduce drop-out in youth sports toward a more inclusive social experience incorporating education in the school context and the key role of family support, as highlighted by the other contributions to this Research Topic.

Further research is certainly needed to better understand how some of the research questions raised in this Research Topic may influence each other and in different contexts and age groups, with the common goal of contributing to a more positive and educational experience of sports practice by the younger generation.

It is indeed advisable to create an explicit network that includes all the social components of this complex phenomenon including children and adolescences, parents and family, school teachers, community representatives, sports coaches and instructors, pedagogues and psychologists and kinesiologists and PA experts, who can act as a link between all these actors involved.
